# The mammalian skull: development, structure and function

**DOI:** 10.1098/rstb.2022.0077

**Published:** 2023-07-03

**Authors:** Łucja Fostowicz-Frelik, Z. Jack Tseng

**Affiliations:** ^1^ Department of Organismal Biology and Anatomy, University of Chicago, Chicago, IL 60637, USA; ^2^ Institute of Paleobiology, Polish Academy of Sciences, 00-818 Warsaw, Poland; ^3^ Department of Integrative Biology, University of California, Berkeley, CA 94720-3200, USA; ^4^ University of California Museum of Paleontology, Berkeley, CA 94720, USA

**Keywords:** mammals, evolution, development

## Abstract

The mammalian skull is an informative and versatile study system critical to research efforts across the broad spectrum of molecular, cellular, organismal and evolutionary sciences. The amount of knowledge concerning mammalian skull continues to grow exponentially, fuelled by the advent of new research methods and new material. Computed microtomography, including X-ray imaging using synchrotron radiation, proved to be an important tool for the descriptive and quantitative analysis of cranial anatomy. A major conceptual change, namely combining genetics and development with evolution into ‘evo-devo’ studies, also contributed to our knowledge of the mammalian skull enormously. These advances, coupled with novel techniques now allow researchers to integrate the process of cranial development with data from the fossil record, which is also augmented by seminal discoveries from Africa, Asia and both Americas. However, for decades, there has been no comprehensive source covering fundamental aspects of this vibrant field of evolutionary biology. To address this gap, we offer in this theme issue a balanced mix of research papers and reviews from leading experts in the field and a younger generation of scientists from five continents.

This article is part of the theme issue ‘The mammalian skull: development, structure and function’.

## 1. Introduction

The mammalian skull, an anatomical entity and a well-distinguishable morphological unit is one of the best examples of how modern morphological research intercalates with other fields of biology and how discoveries from these other disciplines enhance and fulfil one another. The thematic volume we present herein *per force* aims in indicating only the major directions of the research related with this crucial structure in mammals. However, the amount of knowledge concerning mammalian skull continues to grow exponentially, fuelled by the advent of new research methods and new material. On the one hand, computed microtomography has become a central tool for the descriptive and quantitative analysis of cranial anatomy, including even minuscule structures and cranial endocasts. On the other hand, major conceptual change, namely combining development with evolution into ‘evo-devo’ studies, accelerates our knowledge of the mammalian skull enormously. These advances now allow researchers to integrate the process of cranial development with data from the fossil record, which is also augmented by seminal discoveries from Asia, Africa and both Americas. And yet, since the synthetic ‘Mammalian Skull’ by Moore [1], there has been no comprehensive source covering fundamental aspects of this topic.

**Figure 1.**
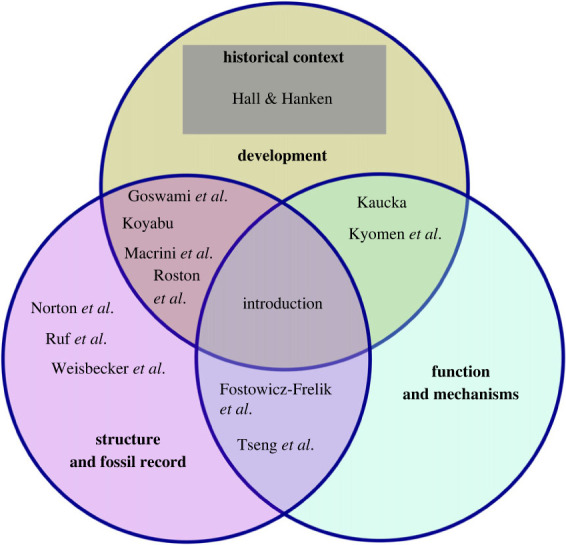
Relationships between contributions within the three conceptual domains of this theme issue.

This endeavour seems all the timelier because 50 years elapsed in 2022 since the death of Professor Sir Gavin de Beer FRS (1899–1972). One intended goal of our theme issue is to celebrate his monumental contribution, *The development of the vertebrate skull* [2], which had a huge impact on the studies of mammalian skull in particular. Below we highlight some of the key topics and take-home messages covered by the contributions included in this issue. The thematic issue is divided into broad subject areas devoted to growth and development, morphology, and function of the mammalian skull (figure 1). We deliberately omit the dentition since its study is a vast discipline in itself. We also did not explore a rapidly growing field of brain endocast research as it merited a separate monographic treatment recently [3].

The main objective of compiling this thematic collection is to offer a snapshot of current developments in the field from multiple rich perspectives, rather than an obviously impossible synthesis of all that is known on the subject. In particular, we strived to highlight developments in three areas that are of primary importance to our understanding of the mammalian skull. These concern the development of the head, the evolution of skull diversity and the functional aspects of skull morphology. Certainly, many of these contributions are cross-disciplinary, thus fitting into more than one category.

## 2. A bit of history

If one were to choose a particular date as to when a new approach (i.e. other than descriptive) to the mammalian skull started, we might propose 1807 for a good reason. That year (9 November, to be exact) Lorenz Oken (1779–1851) delivered an inaugural lecture at the University of Jena entitled ‘On the importance of cranial bones’ [4]. As the story goes, during his excursion in the Harz Mountains, Oken came across the skull of a doe and in the moment of illumination hypothesized that it represented a highly modified spine. That was an early concept connecting comparative anatomy and development. Oken's theory of the skull origin from the modified vertebrae (vertebral theory of the skull) supported in the United Kingdom by the influential Richard Owen FRS (and opposed by Thomas H. Huxley FRS [5] in his Croonian Lecture in 1858), was profoundly revised by Carl Gegenbaur, who, while a professor in Jena, proposed ‘the segmentation theory’ of the vertebrate head [6]. From Gegenbaur to Sir Edwin Ray Lankester FRS (who visited Gegenbaur in Jena) to Edwin Stephen Goodrich FRS, one can eventually reach in this intellectual genealogy Sir Gavin Rylands de Beer FRS. The latter’s first significant contribution to mammalian skull research was *The development of the skull of the shrew* [7] communicated by his mentor Professor Goodrich in the same journal of which we have now the pleasure to edit a volume. The role and influence of Gavin de Beer have been aptly presented in Hall & Hanken [8].

## 3. Development and evolution

As Wanninger [9] pointed out, morphology not only is very much alive but also it is a crucial part of a MorphoEvoDevo approach (i.e. evolutionary developmental morphology). Integrated with phylogenetics, it leads to deep and novel insights, particularly concerning the mammalian skull, as evidenced below.

Our volume includes some examples of such studies. Starting with a more specialized topic, Macrini *et al*. [10] present an ontogenetic development of the turbinals in a wallaby, one of the non-model animals, addressing the problems of homology of the turbinals (nasal region) across the diversity of extant Marsupialia. Ruf *et al*. [11] analyse the course of the internal carotid artery in living and extinct Lagomorpha, teasing out the evolutionary transformations between the stem and crown taxa, and exploring the diversity across nearly all of living genera. Norton *et al*. [12] provide the broadest evolutionary landscape; they explore evolutionary accretion of mammalian characters through the analysis of the cranial structure in cynodonts and mammaliaforms. That reminds us that mammals are synapsids and their skull structure is deeply rooted in the Paleozoic, about 320 Ma [13]. All three contributions show changes in the cranial structures from a phylogenetic perspective, involving time as an instigating factor.

The broad overview by Koyabu [14] explores the osteological variability of the mammalian skull, highlighting ongoing challenges to understanding the homology, ossification patterns, bone fusion, morphology of the sutures and the phenomenon of the neomorphic bones, all from the evo-devo perspective. The contribution by Kaucka [15] is a survey of the craniofacial development regulation and plasticity, exploring genetic patterning during the development of the cranial diversity across mammalian species. The review paper by Kyomen *et al*. [16] focuses on the molecular control of the processes of evolutionary heterochrony, heterotopy and heterometry in mammalian skull, guiding the reader through the intricacies of the shape variability and cranial adaptive modifications in mammals.

## 4. Structure, function and their context

The bones of the mammalian skull form both an integrated biomechanical system and a puzzle of elements of varied phylogenetic and developmental origins, many shared with other vertebrates. However, mammal skulls exhibit reduced cranial kinesis relative to other vertebrates, but conserved pharyngeal arch-derived growth centres intimately link the evolutionary developmental origins of the skull's constituents to their gnathostome ancestors. This duality has sparked a rich body of scientific inquiry into the structure and function of the namesake structure of this theme issue.

On the topic of mammalian skulls structure and function, this theme issue has a collection of five contributions that touch on a wide spectrum of research questions and model clades. On the homology of the skull roof bony elements, Roston *et al*. [17] clarify the evolution and variation in interparietal elements using cetaceans, a clade showcasing arguably the most modified skulls among mammals. On the paradigm of using basicranial characteristics in mammalian systematics, Weisbecker *et al*. [18] demonstrate that, in fact, the basicranium may contain lower degrees of phylogenetic signal than whole skull shape characteristics. Along a similar vein of intracranial variation in evolutionary tempo and mode, Goswami *et al*. [19] show through a deep-time and high-density landmark-based shape analysis of the placental cranium, that different evolutionary rates characterize skull bone elements of different developmental origins, even if the overall disparity of those elements are comparable. Two other contributions also focus on the functional morphology of a relatively less structurally complex, but equally data rich, region of the skull—the mandible. Fostowicz-Frelik *et al*. [20] analysed the cross-sectional mechanical attributes of the mandibular ramus in early diverging Glires and demonstrate the presence of a diverse repertoire of jaw mechanics at the base of Glires prior to the subsequent divergence and radiation into stereotypical rodents and lagomorphs, respectively. In a broad comparison between mammalian mandibles and those of non-mammalian jawed vertebrates, Tseng *et al*. [21] propose the presence of a fundamental shift in the evolvability and structure–function linkage relationship coinciding with the evolutionary transformation from multi-element to a single-element lower jaw in mammals.

These complementary studies on the structural and biomechanical evolution of mammalian skull elements reveal a set of general principles that may guide our ongoing and future collective research efforts into this exceptional biological model system:

— Skull function may be ‘blind’ to evolutionary homology; bony element shape may not necessarily correlate with functional evolutionary trends. Mammalian skull elements and regions may evolve faster or slower based on conserved or novel function selected for at a level different from the immediate identity of the bony elements themselves.
— Even functionally relevant overall skull shape may inform phylogeny better than slow-evolving regions such as basicrania; discrete character and shape characters may reflect different hierarchical levels and timescales of macroevolution.
— Glires, representing the majority of mammals, show that a decoupled incisor versus molar partition led to two fundamentally different biomechanical configurations in the mandible. Such a bi-modal dental functional morphology eventually split into lagomorph and rodent morphotypes. The fact that stem Glires already exhibit biomechanical profiles reminiscent of their extant counterparts suggests that the biomechanical canalization of rodents and lagomorphs into different generalized jaw mechanics blueprints may have had an overarching and yet seldom studied influence on their dramatic differences in diversity.
— Across the placental skull, evolutionary rate, but not disparity, correlates with developmental modules. Evolutionary rates are also associated with dietary and locomotor groups, but operating on a more general level that does not show consistent element-ecology associations. The integration-modularity patterns of the mammalian skull are likely produced under the interaction of these multi-level rate difference generators.
— Rather than evolutionarily releasing mammals into an adaptive radiation of both structure and function, the evolution of the single-element lower jaw, concomitant with the definitive mammalian middle ear, canalized mammalian jaw biomechanics towards a principally stiffness-mediated regime. Despite this low mechanical disparity, mandibular shape in mammals is comparable to or exceeds the disparity observed in other non-mammal vertebrate groups as a whole. This represents a switch in the structure–function configuration of mammalian lower jaws at the base of the crown mammal radiation.

## 5. Concluding remarks

It has been more than four decades since the first edition of *The mammalian skull* (1981) by W.J. Moore was published [1]. In that seminal work, the scientific discussion of the mammalian skull centred around four major sections: the bony elements, their evolution, functional morphology and development. In the intervening years, much of these major directions of research have been augmented by subsequent generations of evolutionary biologists. Perhaps the most substantial shift from Moore's work has been the integration of multiple disciplines within biology and the advent of new imaging and genetic technologies as a nexus of research into all aspects of the mammalian skull. No better example of such a transformation in our discipline can be found than in the content and author list of this thematic issue.

Finally, because we have in mind the famous and anonymous adage saying that it is difficult to make predictions, especially about the future, we hope that this theme issue will spark discussions and collaborations among the students of mammalian skull, inspiring future researchers for the next four decades and beyond.

## Data Availability

This article has no additional data.
